# E6-associated transcription patterns in human papilloma virus 16-positive cervical tissues

**DOI:** 10.3892/ol.2014.2698

**Published:** 2014-11-11

**Authors:** KEZHI LIN, XULIAN LU, JUN CHEN, RUANMIN ZOU, LIFANG ZHANG, XIANGYANG XUE

**Affiliations:** 1 Medical Experimental Teaching Center, Wenzhou Medical University, Wenzhou, Zhejiang 325035, P.R. China; 2 Department of Microbiology and Immunology, Institute of Molecular Virology and Immunology, Institute of Tropical Medicine, Wenzhou Medical University, Wenzhou, Zhejiang 325035, P.R. China; 3 Department of Pathology, Zhuji People’s Hospital of Zhejiang Province, Zhuji, Zhejiang 311800, P.R. China; 4 Department of Obstetrics and Gynecology, First Affiliated Hospital of Wenzhou Medical University, Wenzhou, Zhejiang 325035, P.R. China

**Keywords:** HPV-16, cervical cancer, amplification of papillomavirus oncogene transcripts, papillomavirus

## Abstract

The change in transcription pattern induced by post-transcriptional RNA splicing is an important mechanism in the regulation of the early gene expression of human papilloma virus (HPV). The present study was conducted to establish a method to specifically amplify HPV-16 E6-associated transcripts. The E6-related transcripts from 63 HPV-16-positive cervical tumor tissue samples were amplified, consisting of eight cases of low-risk intraepithelial lesions, 38 cases of high-risk intraepithelial lesions and 17 cases of cervical cancer (CxCa). The appropriate amplified segments were recovered following agarose gel electrophoresis, and subjected to further sequencing and sequence alignment analysis. Six groups of E6 transcription patterns were identified from HPV-16-positive cervical tumor tissue, including five newly-discovered transcripts. Different HPV-16 E6-associated transcription patterns were detected during the development of CxCa. Over the course of the progression of the low-grade squamous intraepithelial lesions to CxCa, the specific HPV-16 E6-associated transcription patterns and the dominant transcripts were all different. As indicated by this study, the transcription pattern of the E6 early gene of HPV-16 was closely associated with the stages of cervical carcinogenesis, and may also be involved in the development of CxCa.

## Introduction

CxCa is the second most common form of malignant tumor that results in female mortalities worldwide. Persistent infection with high-risk human papilloma viruses (HPVs), particularly HPV-16 and HPV-18, is a key factor in the development of CxCa (1). The incidence of high-risk HPV strains has been identified in up to 99.7% of cervical squamous cell carcinomas, and 94–100% of cervical adenocarcinoma and adenosquamous carcinomas (2,3). The expression of viral E6 and E7 oncoproteins by host cells during HPV infection is critical for the induction of CxCa. The E6 protein abrogates p53 function primarily through the binding of ubiquitin-like enzyme E6-associated protein, while the E7 protein inactivates the retinoblastoma protein and p130. Together, these changes result in malignant transformation (4,5). In addition, E6 and E7 expression may be associated with host genome instability (6).

Bicistronic or polycistronic transcription, with two or more open reading frames (ORFs), is a feature of HPVs. Regulatory elements (including E2-binding elements or those that bind to activator protein-1 and octamer-binding protein-1 transcription factors), early promoters (including the p97 of HPV-16, and p99 of HPV-31), early splice sites and early polyadenylation signal sites (7,8), are all involved in the regulation of HPV early gene expression. HPV early gene transcripts are always concomitant with post-transcriptional RNA splicing processes to eliminate non-coding introns from transcripts (7). Different RNA splicing patterns may produce different transcripts, resulting in the production of diverse proteins (7,8). Furthermore, the course of CxCa is always concomitant with HPV integration. This integration into the host genome can considerably change the expression pattern of HPV genes due to the effect of transcriptional and post-transcriptional regulation. Moreover, HPV genome integration may also affect the function of cellular genes through the activation of oncogenes, or the inactivation of tumor-suppressor genes.

While HPV-16 early gene transcription patterns have been reported in recent years, HPV-16 E6 transcription patterns, during the course of CxCa genesis have not been systematically analyzed. Therefore, the present study used the amplification of papillomavirus oncogene transcripts (APOT) to determine the profiles of E6-associated early gene transcripts and comprehensively investigate the variation of transcriptional patterns in eight low-grade squamous intraepithelial lesions (LSILs), 24 high-grade squamous intraepithelial lesions (HSILs) and eight CxCa HPV-16-positive cervical biopsy samples.

## Materials and methods

### Patients and specimens

Samples from 63 hospitalized or outpatient subjects, with HPV-16-positive cervical malignancies, were collected from the Second Affiliated Hospital of Wenzhou Medical University (Wenzhou, Zhejiang, China) between December 2010 and April 2012. These samples included eight cases of LSILs, 38 cases of HSIL consisting of 22 cervical intra-epithelial neoplasia (CIN) II cases and 16 CIN III cases, and 17 CxCa cases. The patient age range was 25–59 years, with a median age of 43 years. HPV genotyping and pathological confirmation was conducted for all specimens during the collection. The patients did not receive any radiotherapy or chemotherapy prior to the surgery. In addition, eight control cervical tissue samples negative for HPV and with normal cytology were obtained from patients who underwent a hysterectomy owing to benign gynecological diseases. Subsequent to *ex-vivo* procedures, the specimens were preserved in liquid nitrogen until further experimentation. This study was approved by the Medical Ethics Committee of the First Affiliated Hospital of Wenzhou Medical University (Wenzhou, Zhejiang, China). All participating patients provided informed consent.

### RNA isolation

The cryopreserved cervical tissues were crushed to a powder in liquid nitrogen, and the RNA was extracted with TRIzol reagent according to the manufacturer’s instructions (Invitrogen, Carlsbad, CA, USA). To remove the residual contaminating DNA, the RNA preparation was treated with RNase-free DNase I (Takara Biotechnology, Co., Ltd., Dalian, China). The purified RNA was dissolved in RNase-free water (Toyobo Co., Ltd. Osaka, Japan) and stored at −80°C. The concentration and purity of total RNA were quantified by an ultraviolet spectrophotometer (DU640, Beckman Coulter, Miami, FL, USA) at 260 and 280 nm, respectively, and the integrity of the RNA was examined by 1% agarose gel electrophoresis. Only RNA samples with an A260/A280 ratio of 1.8–2.0 and high integrity were used for further experiments.

### Reverse transcription and amplification of oncogene transcripts

The APOT assay was used to amplify HPV oncogene transcripts. The total RNA (1 μg) was reverse transcribed using an oligo (dT)_17_ RT primer, coupled to a linker sequence (5′-GAC TCGAGTCGACATCGATTTTTTTTTTTTTTTTT’3) (9). Reverse transcription was conducted for 1 h at 42°C in a reaction system containing 2.5 μM RT primer, 200 units of Moloney murine leukemia virus reverse transcriptase (Toyobo Co., Ltd.), 20 units of RNase Inhibitor (Toyobo Co., Ltd) and 1X RT buffer, in a final volume of 20 μl. In order to open the RNA stem-loop structures, the RNA samples were incubated at 65°C for 10 min prior to mixing with reaction buffer and reverse transcriptase. The cDNA obtained was subsequently amplified by polymerase chain reaction (PCR) in a total volume of 50 μl, using 1 unit of KOD-plus DNA Polymerase (Toyobo Co., Ltd), 0.2 μM HPV-16 E6-specific forward primer P1 (5′-CGACCCAGAAAG TTACCAC-3′) and 0.2 μM P0 reverse primer (5′-GACTCGAGT CGACATCGA-3′). The reaction mixture was subjected to an initial denaturation step for 90 sec, followed by 35 cycles of denaturation at 94°C for 30 sec, annealing at 59°C for 30 sec, elongation at 68°C for 2 min, and a final elongation step at 68°C for 6 min to ensure the integrity of the amplified fragments.

### Southern hybridization

The final PCR products were electrophoresed in 2.5% agarose gels, and the DNA was transferred to a nitrocellulose membrane (Amersham Life Sciences, Buckinghamshire, England) and fixed for 2 h at 80°C prior to mixing with hybridization solution. Following pre-hybridization for 1 h, the membranes were incubated overnight at 46°C in hybridization solution, with a 5′-biotin-labeled HPV-16 E6-specific probe (5′-CTGCGACGTGAGGTATAT GACTTTG-3′) at a final concentration of 100 ng/ml (Thermo Scientific, Co., Ltd., Waltham, MA, USA). Following hybridization, the membranes were washed twice with pre-warmed 2X saline sodium citrate (SSC) solution containing 0.1% sodium dodecyl sulfate (SDS) at 50°C for 20 min, and twice with pre-warmed 0.2X SSC solution containing 0.1% SDS at 53°C for 15 min. Detection of the probe signal was performed using a chemiluminescence detection system (Thermo Scientific, Co., Ltd.) according to the manufacturer’s instructions, followed by film exposure.

### Sequence analysis of HPV-16 E6-associated transcripts

The APOT amplification products were visualized by 2.5% agarose gel electrophoresis. The PCR-amplified fragments of interest were separated and purified from the gel using an agarose gel DNA extraction kit, according to the manufacturer’s instructions (TianGen, Beijing, China). The corresponding amplimers were cloned into a *pEASY*™-blunt zero cloning vector (TransGen, Beijing, China), and the sequencing of amplified sequences was conducted using an ABI3730 XL Genetic Analyzer (Applied Biosystems, Waltham, MA, USA), according to standard procedures. The alignment analysis of the sequencing results was conducted using the BLASTn service supplied by the National Center for Biotechnology Information (Bethesda, MD, USA).

## Results

### Establishment of specific amplification for HPV-16 E6-associated transcripts

**Figure 1:**
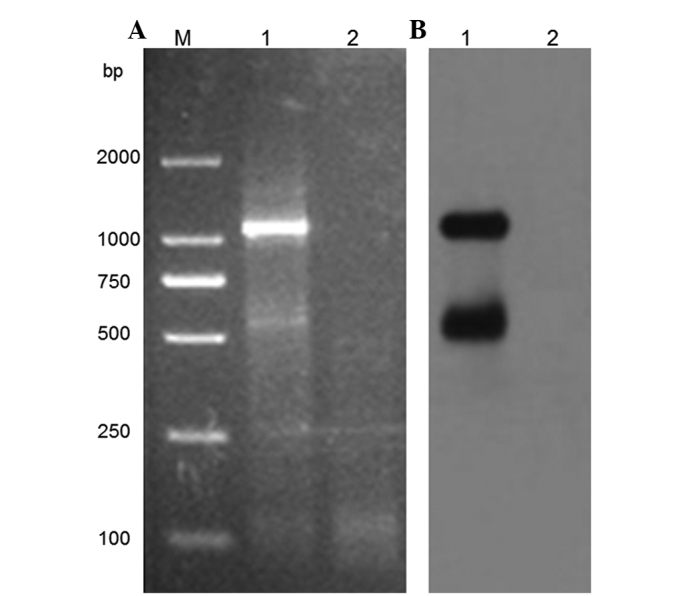
Specificity analysis of the amplification of papillomavirus oncogene transcripts assay for human papilloma virus (HPV)-16 E6-associated transcripts. (A) 2% agarose gel electrophoresis and (B) E6-specific probe Southern blot demonstrating the presence of HPV-16 E6-associated transcripts in Caski cells. M, D2000 marker; lane 1, Caski cells; lane 2, normal cervical tissue.

The basis of an APOT assay is the 3′ rapid amplification of cDNA ends, which enables amplification and cloning of the region between a single short sequence in the cDNA molecule, and its unknown 3′ end (10). In order to analyze HPV-16 E6-associated transcripts, mRNA from tissue samples was reverse transcribed to cDNA, using an RT primer containing a conservative sequence in its 5′ end. PCR amplification was conducted using HPV-16 E6-specific (P1) and -conserved (P0) sequences, which resulted in amplification and cloning of the region between a single short sequence in a cDNA molecule and its unknown 3′ end (10). This primer design amplified the E6-associated transcripts from episomal and integrated viral genomes. To verify the specificity of the modified APOT assay, cDNA from HPV-16-positive Caski cells, containing integrated HPV-16 genome, and HPV-negative normal cervical tissues, were used. Fig. 1A demonstrates that the target amplimer could be detected in the HPV-16-positive Caski cells, whereas it was absent in the normal cervical tissue samples. The presence of HPV-16 E6-associated transcripts was further confirmed by southern blotting using HPV-16 E6 probes (Fig. 1B), which suggests that this method may specifically amplify the HPV-16 E6-associated transcripts.

### Characteristics of the HPV-16 E6-associated transcriptional pattern in the tissues of CIN and CxCa

**Figure 2:**
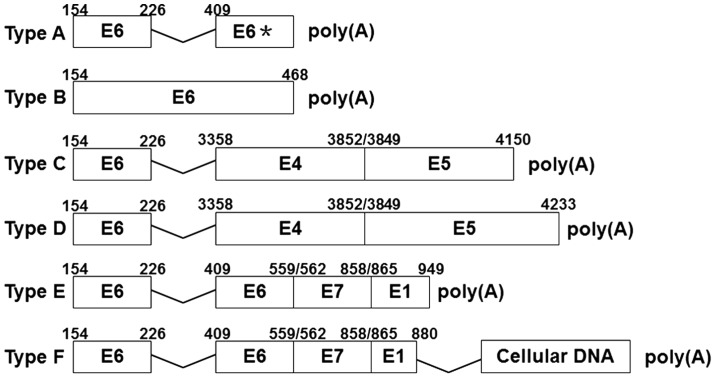
Schematic diagram of the HPV-16 E6 transcription patterns (types A–F) identified in cervical cancer tissues. ^*^Representing two categories of disconnection sites at nucleotides 442 and 468.

**Figure 3:**
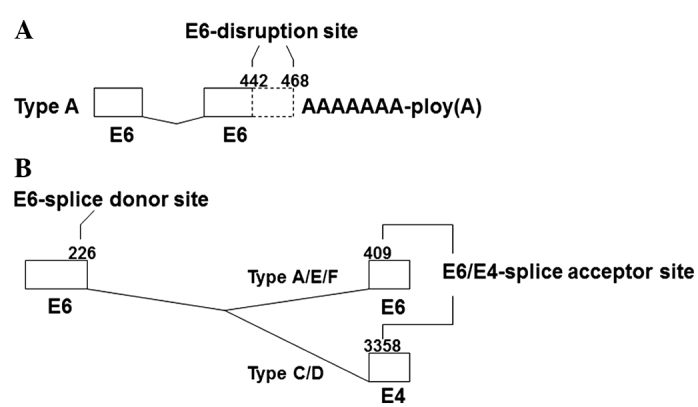
Schematic diagram of (A) two disconnection patterns and (B) two splicing patterns.

To identify HPV-16 E6-associated transcripts, 63 RNA samples of good quality were obtained from HPV-16-positive cervical specimens (LSIL, n=8; HSIL, n=38; CxCa, n=17), and were amplified by PCR according to the aforementioned method. Six different types of HPV-16 E6-associated transcription patterns were identified (Fig. 2). Among these patterns, types A, B, C, D and E were all directly connected with the polyadenylation site and lacked host-cell genetic material. Furthermore, transcription patterns C and D ended with an unbroken E5 (the total sequence of E5), and were affected by an early polyadenylation signal, which suggests that they are episomal patterns. Meanwhile, the A, B and E transcripts contained untraditional splice donor signals at nucleotides (nts) 442, 468 and 949, suggesting that they are potential integration transcripts (Figs. 2 and 3A). The type F transcripts were connected with host genome sequences, clearly identifying them as integration transcripts. In pattern A, the HPV-16 E6 gene was spliced in the sites of nt 226, a splice donor signal, and nt 409, a splice acceptor signal (Figs. 2 and 3B). In addition, there were different disruptions at the 3′ end of the E6 gene region, located at nt 442 and 468 (Fig. 3A). In pattern B transcripts, the site of disruption was located at nt 468 of the HPV-16 E6 gene, with no internal splicing observed. Patterns C and D contained the same gene fragments of partial HPV-16 E6, E4 and E5 genes, with splice sites at nt 226 in E6, and nt 3,356 in E4 (Fig. 3B). However, the sequence of E5 at their 3′ ends was different. Patterns E and F also contained the same HPV-16 early gene fragments, with E6 internal splicing. However, in pattern F, the HPV-16 E1 gene was disrupted at nt 880, and was connected with host genome sequences. With the exception of pattern F, all the HPV-16 E6-associated transcript patterns were first reported by Wentzensen *et al* (11). Of these six HPV-16 E6-associated transcripts, patterns E and F accounted for 53.8%.

### Changes in the HPV-16 E6 transcription patterns during carcinogenesis and the development of CxCa

**Figure 4:**
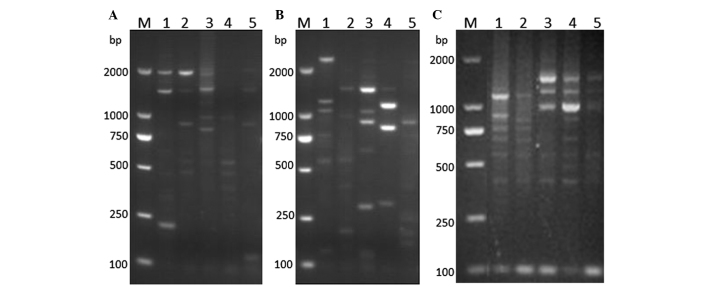
HPV-16 E6-associated transcription pattern analyses for low-grade squamous intraepithelial lesions (LSIL), high-grade squamous intraepithelial lesions (HSIL) and cervical cancer (CxCa) tissues. (A) LSIL (B) HSIL and (C) CxCa. M, D2000 marker; lanes 1–5 represent five different samples in each pathological type.

**Figure 5:**
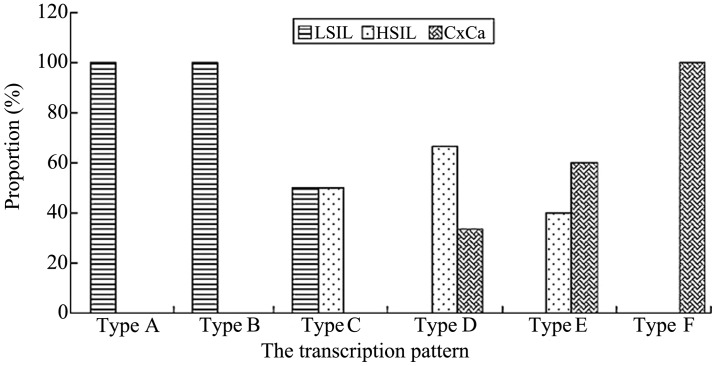
Results demonstrating that the proportions of six different HPV-16 E6-relevant transcription patterns (types A–F) alter during the progression from low-grade squamous intraepithelial lesions (LSIL), to high-grade squamous intraepithelial lesions (HSIL), to cervical cancer (CxCa).

HPV-16 E6-associated transcriptional patterns were systematically analyzed in HPV-16-positive tissues of LSIL, HSIL and CxCa, and were found to significantly differ between the different tissues. Over the course of LSIL progression to CxCa, there was an observable difference in the expression of specific HPV-16 E6-associated transcription patterns (Fig. 4 and 5). All samples positive for patterns A and B were identified in cases of LSIL, while all samples positive for pattern F were identified in cases of CxCa (Fig. 5). In total, pattern C accounted for 50% of the transcripts in LSIL and HSIL. Overall, 66.67% of cases of HSIL were positive for pattern D transcripts. However, pattern D represented only 33.33% of the transcripts in CxCa, and was absent in LSIL. In total, 60% of pattern E transcripts were detected in CxCa samples, with the rest identified in HSIL specimens.

## Discussion

The double-stranded, circular DNA genome of HPV is ~7.9 kb in length and consists of the non-coding long control region, the early gene region (encoding the early genes of E1, E2, E4, E5, E6 and E7) and the late gene region (encoding the capsid proteins L1 and L2) (7). Due to the polycistronic characteristics of the HPV genome, different profiles of HPV gene expression may result from different transcriptional patterns and the splicing of these transcripts (7). The transcriptional pattern of episomal HPV-16 infection has been previously elucidated (7), and it is recognized that the HPV genome integrates into the host cell genome in cervical carcinogenesis, which may alter the transcription pattern of HPV genes (9). The majority of previous studies have focused on E7-associated transcriptional patterns. Several E7-associated transcriptional patterns, including the episomal type E7-E1^E4 (where ‘^’ represents splicing), and the integrated types E7-E1^-cellular and E7-E1^E4-cellular DNA, were revealed in HPV-16-positive CxCa (9,11). In addition to the HPV E6 full-length transcript, two E6-associated transcripts, termed E6*I and E6*II (where ‘*’ represents the splice site), have been previously reported (7). The E6*I and E6*II transcripts use the site at nt 226 in the E6 ORF region as the donor splice site, and the sites of nt 409 and nt 526 in the HPV-16 genome as the splicing receptor sites. Previous studies have demonstrated that the E6*I and E6*II patterns are associated with the promotion of E7 expression (12,13). In HPV-16-positive CxCa tissues, additional E6-associated transcription patterns, including integrate-derived transcripts of E6-E7-E1, E6-E7-E1^E4, E6-E7-E1 (integrated E1 gene), E6-E7-E1 (disintegrated E1 gene) and an episomal transcript of E6-E7-E1^E4, have also been identified (11,14). However, the presence of these transcriptional patterns, and their correlation with carcinogenesis and the development of CxCa, have not yet been investigated.

In order to analyze the HPV-16 E6-associated transcription profile in the different stages of CxCa, an APOT assay was used in the present study to determine E6-associated transcript patterns in 63 HPV-16-positive cervical tumor tissues. In total, six E6-associated transcription patterns were identified in the HPV-16-positive cervical tumor tissues. In contrast with previous studies (11,14,15), the present study described five transcriptional patterns (A, B, C, D and E) for the first time using the APOT method, but did not identify a complete E6 gene. A potential reason for this is that the APOT assay may not be sufficient in amplifying long integrate-derived transcripts. In addition, it tends to amplify those transcripts present at higher levels, and exclude those expressed at lower levels (16). Alternatively, post-transcriptional splicing may contribute to these results (7,12,17). It was also observed that the integrated E7 gene was preserved in the E6-associated transcripts of patterns E and F. Therefore, E6 splicing may also contribute to HPV-16 E7 expression, as previously reported for E6*I and E6*II transcripts (7).

The role of HPV-16 early gene transcription patterns in the carcinogenesis and development of CxCa has not yet been established by previous studies. In the present study, distinct differences in HPV-16 early gene transcriptional patterns, at different stages of CxCa progression, were observed. All samples positive for patterns A and B originated from patients with LSIL, while all samples positive for pattern F were identified in patients with CxCa. In total, 50% of the samples from patients with LSIL and HSIL were positive for pattern C transcripts, while the majority of samples positive for patterns D and E were identified in patients with HSIL and CxCa. Van Tine *et al* (18) proposed that the evolution of transcription patterns may be a dynamic process in response to environmental changes, to minimize gene expression associated with cell proliferation (18). This may also explain the existence of different HPV-16 E6-associated transcriptional patterns with the progression of CxCa.

## Abbreviations

HPV: human papilloma virus
ORFs: open reading frames
APOT: amplification of papillomavirus oncogene transcripts
HSILs: high-grade squamous intraepithelial lesions
